# Single-site laparoscopic colectomy for rectosigmoid cancer with middle aortic syndrome: report of a case

**DOI:** 10.1186/s40792-015-0050-4

**Published:** 2015-06-19

**Authors:** Koki Tamai, Ichiro Takemasa, Mamoru Uemura, Junichi Nishimura, Taishi Hata, Hiroki Higashihara, Keigo Osuga, Tsunekazu Mizushima, Hirofumi Yamamoto, Yuichiro Doki, Masaki Mori

**Affiliations:** Department of Gastroenterological Surgery, Graduate School of Medicine, Osaka University, Yamadaoka 2-2, Suita, Osaka 565-0871 Japan; Department of Diagnostic and Interventional Radiology, Graduate School of Medicine, Osaka University, Osaka, Japan

**Keywords:** Single-site laparoscopic colectomy, Middle aortic syndrome, Colorectal cancer

## Abstract

**Introduction:**

Single-site laparoscopic colectomy (SLC) is a promising minimally invasive and safe treatment for colorectal cancer. Improvements of the working instruments and procedures for SLC have helped to overcome challenges regarding the difficulty of operation, supporting the gradual acceptance of this technique. In contrast, narrow working space of the abdominal cavity sometimes prevents securing an adequate surgical view. To obtain precise anatomical information and enable complete mesocolic excision (CME), we routinely perform three-dimensional computed tomography prior to SLC.

**Case presentation:**

A 69-year-old Japanese woman was clinically diagnosed with rectosigmoid cancer. Unexpectedly, preoperative examination revealed asymptomatic stenosis of the great artery, which was diagnosed as middle aortic syndrome. Because radical colectomy requires dissection of vessels that supply blood flow to the legs, a vascular stent was inserted prior to operation. We chose SLC due to the reduced risk of damaging epigastric arteries, which may eventually become collaterals in the event of stent re-stenosis. We accomplished SLC with CME, and the patient was discharged on the tenth day after operation without complications.

**Conclusion:**

The present case is the first to proceed by SLC for colorectal cancer complicated by vascular obstructive disease. Preoperative imaging enabled us to identify an unexpected rare disease and to still accomplish SLC with CME, thus reinforcing the importance of preoperative imaging to optimize the use of SLC. In addition, SLC may become one of the most adequate procedures for patients complicated by vascular obstructive disease.

## Background

Single-site laparoscopic colectomy (SLC) for colorectal cancer is a minimally invasive surgical procedure that satisfies patients’ demands regarding pain reduction and cosmetic improvement, as well as reduces risks of complications [1]—such as bleeding, port-site hernia, and internal organ damage—which increase with the degree of abdominal wall destruction [2, 3]. However, the capabilities of SLC are limited by instrument collision, loss of triangulation [4], and difficulty of securing an adequate surgical view. Measures to overcome these limitations include the development of specialized working instruments, improvement of techniques, and obtaining more accurate information by careful preoperative examinations. It is important for colorectal cancers to be removed with complete mesocolic excision (CME); therefore, preoperative examination is essential to determine vascularity, which is difficult to clearly visualize in the surgical field during SLC [5, 6]. We use three-dimensional computed tomography (3D-CT) to preoperatively evaluate the tumor feeding artery and drainage vein.

Here, we report a case in which we discovered an unexpected occlusive disease of a great vessel during computed tomography angiography (CTA) prior to SLC. Using this information, we successfully performed stent placement and then achieved SLC. To the best of our knowledge, this is the first literature of laparoscopic surgery for colon cancer with occlusive disease of great vessels. The present case study highlights the importance of preoperative imaging and the utility of SLC.

## Case presentation

**Fig. 1:**
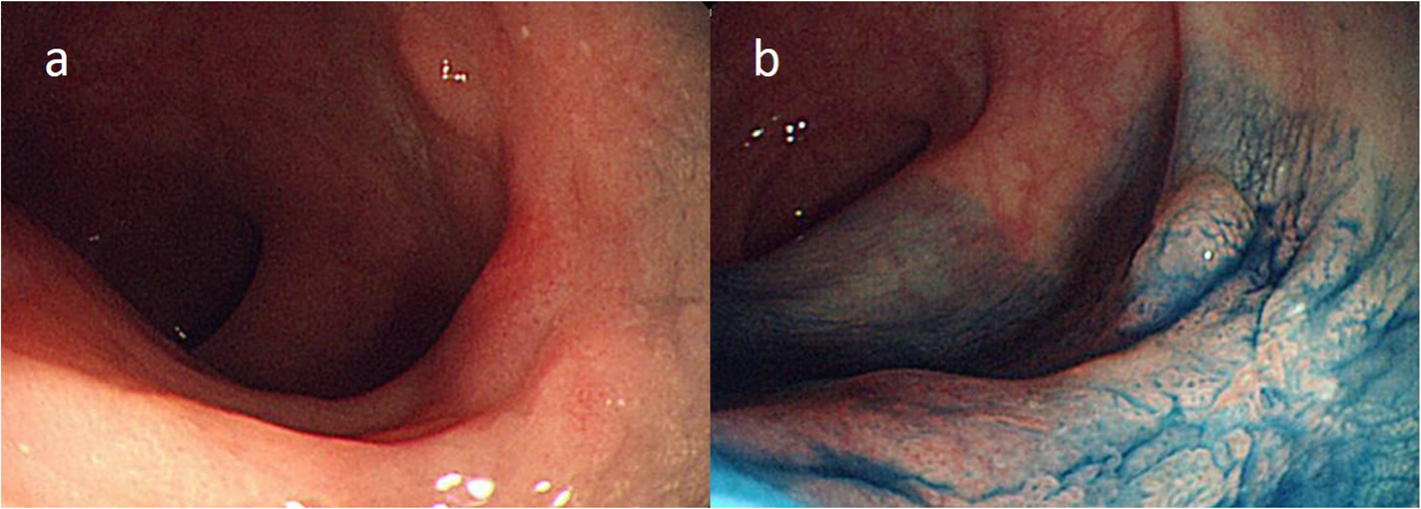
Preoperative colonoscopy findings. **a** Preoperative colonoscopy showed a superficial lesion with central depression in the rectosigmoid colon. **b** Dye dispersion revealed central depression and mucosal unevenness

**Fig. 2:**
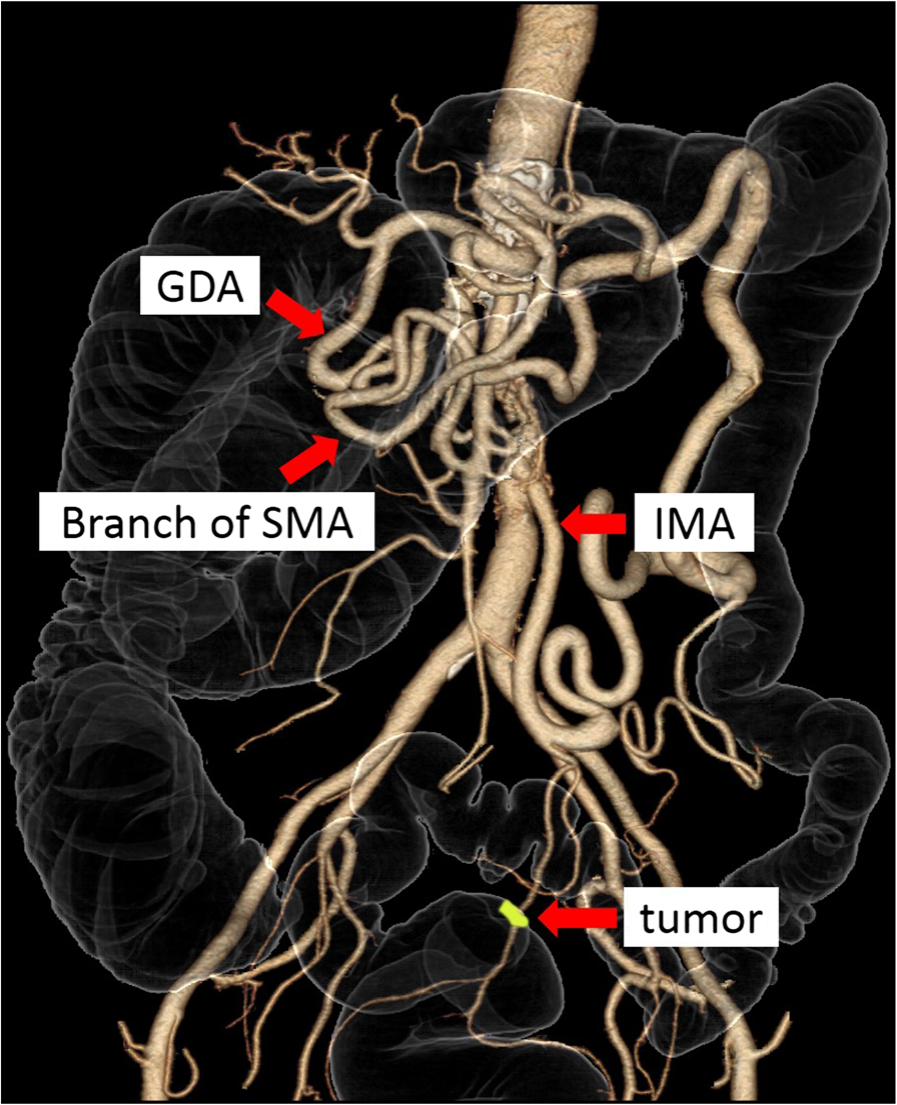
Merged image of computed tomography angiography and colonography. Computed tomography angiography revealed abdominal aortic locoregional stenosis. Accompanying the descending colon, collateral circulations comprised the gastroduodenal artery (GDA) and superior mesenteric artery (SMA), with flow into the inferior mesenteric artery (IMA), and deficiency of the left colic artery

A 69-year-old female Japanese patient suffered from narrow stool and was determined to have a 15-mm superficial lesion with central depression in the rectosigmoid colon (Fig. 1). Invasive carcinoma was suspected and biopsy of the lesion confirmed well-differentiated adenocarcinoma. Positron-emission tomography—computed tomography showed no metastasis. We planned laparoscopic surgery, and CTA was performed to investigate the local vascularity. Unexpectedly, this revealed an approximate 5-cm stenosis from the infrarenal abdominal artery to the root of the inferior mesenteric artery (IMA) and collateral circulations, although the patient had no complaint of angiostenosis symptoms (Fig. 2).

We observed no blood vessel wall thickness at the narrow segment and only a little calcification, then we diagnosed middle aortic syndrome (MAS). Although we did not detect the overswelling of the inferior epigastric artery that is usually seen in this condition as the collaterals to the legs, the IMA communicated with the collateral circulation branched from the superior mesenteric artery and gastroduodenal artery, such that blood flow to below the narrow segment was expected to be due to IMA reflex. Furthermore, deficiency of left colic artery (LCA) meant that the bloodstream to below the narrow segment depended only on collateral circulation, namely, curative colectomy with vascular ligation blocked blood flow to the legs.

**Fig. 3:**
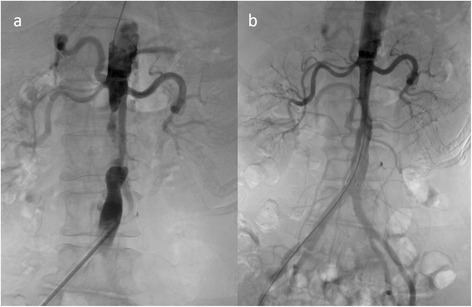
Vascular imaging and endovascular procedure. **a** Obvious segmental stenosis was found at the infrarenal abdominal artery. **b** A self-expandable stent at the region of stenosis secured blood flow to the legs

The patient exhibited no hypertension, leg numbness, coldness, or intermittent claudication—but further examinations revealed the following low ankle brachial index (ABI) values: 0.69 in the right and 0.66 in the left. From this information, we anticipated a low ability to preserve blood flow to the legs and decided that vascular reconstruction was needed. Angiography revealed that the collateral circulation flowed to the IMA, as expected from the CTA (Fig. 3a). We chose to perform stent reconstruction because it is simple and less invasive, and because a properly placed stent would make blood flow obstruction unlikely in the event of obstruction of the IMA root. A self-expandable stent was placed in the narrow segment, and we verified the pressure gradient improvement above and below the narrow segment (Fig. 3b). ABI was improved to 1.02 in the right and 0.95 in the left.

**Fig. 4:**
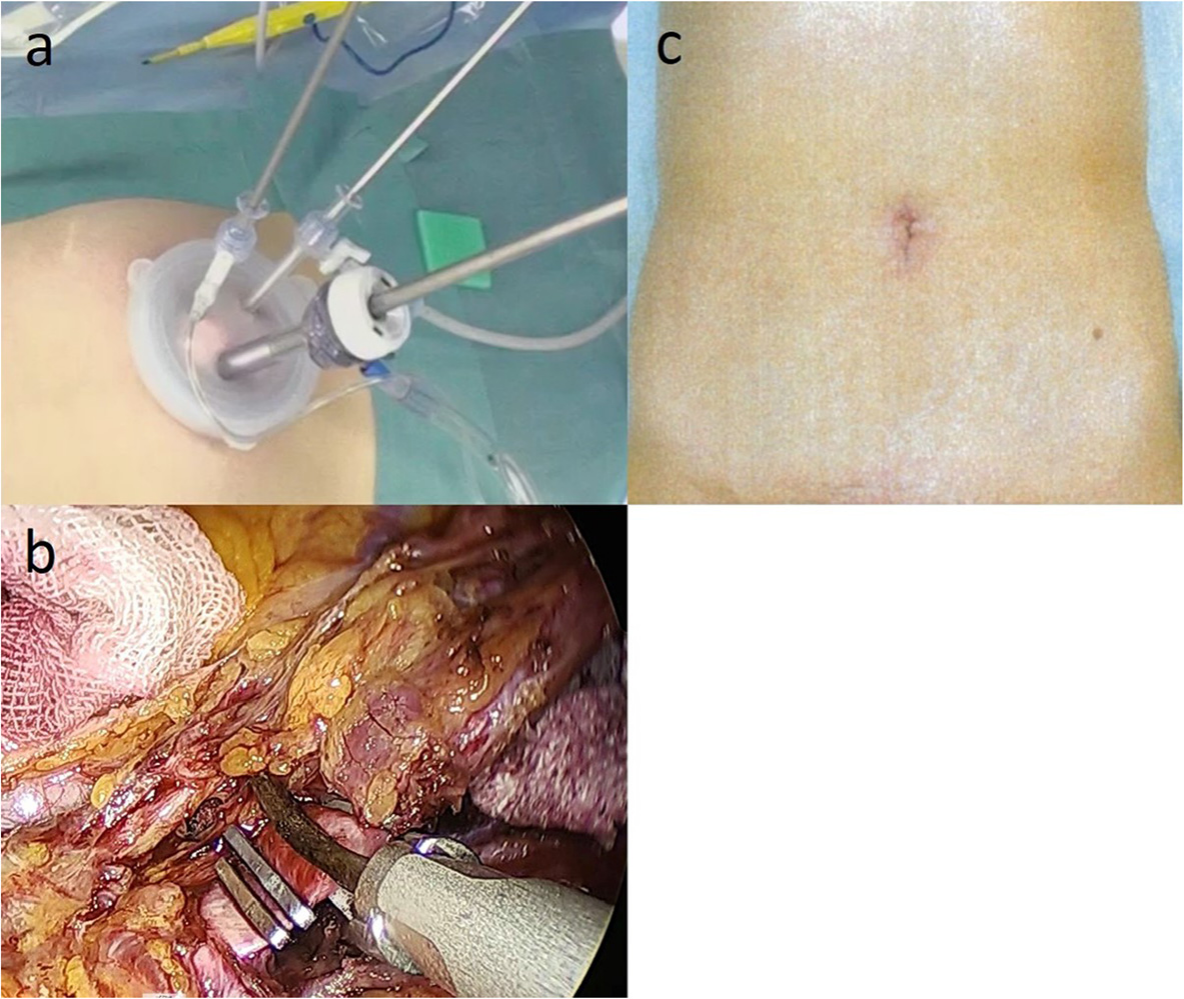
Intra and postoperative view of the abdomen. **a** Photograph of trocar placement. We used an EZ Access (Hakko, Nagano, Japan). **b** Surgical view, showing that IMA was ligated with vascular clip. **c** Postoperative abdominal view, showing that the umbilical incision was not extended and there was no additional lateral abdominal trocar

Eight days after vascular reconstruction, we performed single-incision laparoscopic anterior resection. We started SLC with a 2.5-cm vertical incision in the umbilicus (Fig. 4a). We ligated IMA with a vascular clip and then completed CME (Fig. 4b). The colon was resected with sufficient distal and proximal margins from the tumor and then anastomosed. We did not need additional trocar (Fig. 4c). The patient showed no symptoms of leg ischemia, although ABI fell to 0.88 in the right and 0.77 in the left. The patient was received 20,000 unit/day heparin administration after stenting until 3 h prior to surgery and restarted heparinization 15,000 unit/day 3 days after surgery. Cilostazol and aspirin were started 7 days after surgery; then, the heparinization was ended the next day of administering antiplatelet drugs. The patient was discharged on the tenth day after operation with no complications. Histological examinations revealed the tumor to be moderately differentiated adenocarcinoma with invasion to the submucosa (T1) and no lymph node metastasis (N0). No distant metastasis was found (M0) at the time of surgery, and the histological staging of the tumor was stage I. The root of the IMA was histologically examined to determine whether any vascular lesion existed, revealing slight inner wall thickness and calcification, but otherwise no other remarkable findings.

## Discussion

Many reports describe the minimal invasiveness and safety of SLC for colorectal cancer. SLC has gradually been accepted for its improved patient satisfaction, while also ensuring adequate oncologic clearance and safety [7]. CME with vascular ligation is now advocated for radical resection of colorectal cancer [8]. In a 2014 report of the feasibility of CME with SLC, Takemasa et al. [9] showed that oncologically satisfying surgery can be achieved by SLC. Achieving CME with central vascular ligation requires determination of the precise anatomy. As it is difficult to secure an adequate surgical view during SLC, a detailed preoperative examination is required. Careful preoperative examinations reduce the stress of surgeons and improve operative accuracy.

In the present case, CTA was performed as a preoperative examination prior to laparoscopic surgery, and it unexpectedly revealed stenosis of the abdominal aorta and development of collateral circulations. We diagnosed MAS, which is a rare disease of unknown cause that involves locoregional stenosis between the arch and the terminal bifurcation of the aorta [10]. Although MAS is usually recognized by hypertension caused by renal artery stenosis and leg ischemia, the presently described patient had no related complaints and lacked renal artery stenosis. Generally, asymptomatic MAS is treated conservatively, but here, we performed vascular reconstruction to reduce the possibility of leg ischemia following collateral circulation resection. MAS reconstruction typically involves bypass surgery, but less invasive endovascular therapy has also been reported recently [11]. Here, we chose stent therapy due to the uncomplicated region of stenosis and the need to plan the subsequent laparoscopic colectomy as soon as possible.

Although vascular reconstruction secured the blood flow to the legs, it was also important to avoid damaging the epigastric artery so that it would still be available to serve as collateral circulation in the event of future stent re-stenosis. From the various possible procedures, we chose SLC for several reasons. While laparotomy with midline incision is unlikely to directly damage the epigastric artery, this procedure is highly invasive, and the operator must be careful not to compress the epigastric artery with the wound retractor as this has been reported to obstruct blood flow [12]. On the other hand, multisite laparoscopic colectomy is less invasive than laparotomy, but the port placed in the lateral abdominal region can damage collateral circulations. Overall, SLC with an umbilical incision—which is the thinnest part of the abdominal wall—was determined to carry a low risk of vascular injury and to be less invasive and safer procedure [13]. As planned, we were able to remove the tumor with a fan-shaped excision, including the complete mesentery and domain lymph nodes. In this case, SLC would have been extremely dangerous without a detailed preoperative examination; however, the preoperative information acquired by 3D-CT made SLC the safest operation.

There are only four previously reported cases of operation for colorectal cancer with stenosis of the abdominal aorta and collateral circulation (Table 1). All four previous cases were operated via laparotomy, with the present case being the first to proceed by SLC. Here, the performance of SLC enabled us to deal with the unexpected anomaly, without impairing the effectiveness of operation. Patients suffering from colorectal cancer are in a hypercoagulable state [14]. As vascular obstructive diseases are becoming increasingly common, it is more important to recognize the importance of preoperative imaging prior to SLC for colorectal cancer.

**Table 1:** Literature describing aortic occlusive disease with colorectal cancer

Case	Authors	Years	Sex	Age	Flow to legs	Revascularization	Operation	Approach	Range of LND	Reference
1	Maeda	1987	F	70	IMA-IIA-FA	Thromboendarterectomy	Abdominoperineal excision	Open	NA	[15]
2	Maeda	1987	M	63	CIA	None	Abdominoperineal excision	Open	NA	[15]
3	Itano	1998	M	68	IMA-IIA-FA	Ax-Fa bypass	High anterior resection	Open	D1	[16]
4	Ohara	2008	M	65	CIA-IEA	None	Sigmoidectomy	Open	D1	[17]
5	our case	2014	F	69	IMA-AA-CIA	Stent (abdominal aorta)	Sigmoidectomy	SLC	D3	

*LND* lymph node dissection, *IMA* inferior mesenteric artery, *IIA* internal iliac artery, *FA* femoral artery, *CIA* circumflex iliac artery, *IEA* inferior epigastric artery, *AA* abdominal artery, *SLC* single-site laparoscopic colectomy, *NA* not available

## Conclusions

In the presently described case, preoperative imaging enabled the discovery of an unexpected rare vascular obstructive disease. The information obtained from this preoperative examination was sufficient to enable use to take adequate safety precautions and to still perform curative resection with SLC, without any complications. The present case reaffirms the importance of preoperative diagnosis and indicates the merit of SLC for patients complicated by vascular obstructive disease.

## Consent

Written informed consent was obtained from the patient for publication of this case report and any accompanying images. A copy of the written consent is available for review by the Editor-in-Chief of this journal.

## Abbreviations

3D-CT: three-dimensional computed tomography
ABI: ankle brachial index
CME: complete mesocolic excision
CTA: computed tomography angiography
IMA: inferior mesenteric artery
MAS: middle aortic syndrome
LCA: left colic artery
SLC: single-site laparoscopic colectomy
